# High plasma concentration of non-esterified polyunsaturated fatty acids is a specific feature of severe COVID-19 pneumonia

**DOI:** 10.1038/s41598-021-90362-9

**Published:** 2021-05-24

**Authors:** Maxime Nguyen, Abderrahmane Bourredjem, Lionel Piroth, Bélaïd Bouhemad, Antoine Jalil, Gaetan Pallot, Naig Le Guern, Charles Thomas, Thomas Pilot, Victoria Bergas, Hélène Choubley, Jean-Pierre Quenot, Pierre-Emmanuel Charles, Laurent Lagrost, Valerie Deckert, Jean-Paul Pais de Barros, Pierre-Grégoire Guinot, David Masson, Christine Binquet, Thomas Gautier, Mathieu Blot, Lymphonie study group, Pascal Andreu, François Aptel, Jeremy Barben, Guillaume Beltramo, Philip Bielefeld, Philippe Bonniaud, Bernard Bonnotte, Jean-Baptiste Bour, Marielle Buisson, Pascal Chavanet, Auguste Dargent, Marjolaine Georges, Alexandre Guilhem, Julien Guy, Marie Labruyère, Serge Monier, Suzanne Mouries-Martin, Sébastien Prin, Alain Putot

**Affiliations:** 1 Department of Anesthesiology and Intensive Care, CHU Dijon and University of Burgundy, François Mitterrand University Hospital, LNC UMR1231, 21000 Dijon, France; 2 Univ. Bourgogne Franche-Comté, LNC UMR1231, 21000 Dijon, France; 3 INSERM, LNC UMR1231, 21000 Dijon, France; 4 FCS Bourgogne-Franche Comté, LipSTIC LabEx, 21000 Dijon, France; 5 Inserm, CIC1432, Clinical Epidemiology Unit, Clinical Epidemiology/Clinical Trial Unit, Dijon University Hospital, Dijon, France; 6 Infectious Diseases Department, François Mitterrand University Hospital, Dijon, France; 7 Department of Intensive Care, François Mitterrand University Hospital, Dijon, France; 8 Lipidomic Analytic Platform, University of Burgundy, Dijon, France; 9 Laboratory of Clinical Chemistry, François Mitterrand University Hospital, Dijon, France; 10 Geriatrics Internal Medicine Department, Dijon Bourgogne University Hospital, Dijon, France; 11 Department of Pneumology, Dijon Bourgogne University Hospital, Dijon, France; 12 Department of Internal Medicine and Systemic Diseases, Dijon Bourgogne University Hospital, Dijon, France; 13 Department of Internal Medicine and Clinical Immunology, Dijon Bourgogne University Hospital, Dijon, France; 14 Laboratory of Virology, Dijon Bourgogne University Hospital, Dijon, France; 15 Hematobiology, Dijon Bourgogne University Hospital, Dijon, France; 16 Cytometry Core Facility, University of Burgundy Franche-Comté, Dijon, France

**Keywords:** Infection, Sepsis

## Abstract

COVID-19 pneumonia has specific features and outcomes that suggests a unique immunopathogenesis. Severe forms of COVID-19 appear to be more frequent in obese patients, but an association with metabolic disorders is not established. Here, we focused on lipoprotein metabolism in patients hospitalized for severe pneumonia, depending on COVID-19 status. Thirty-four non-COVID-19 and 27 COVID-19 patients with severe pneumonia were enrolled. Most of them required intensive care. Plasma lipid levels, lipoprotein metabolism, and clinical and biological (including plasma cytokines) features were assessed. Despite similar initial metabolic comorbidities and respiratory severity, COVID-19 patients displayed a lower acute phase response but higher plasmatic concentrations of non-esterified fatty acids (NEFAs). NEFA profiling was characterised by higher level of polyunsaturated NEFAs (mainly linoleic and arachidonic acids) in COVID-19 patients. Multivariable analysis showed that among severe pneumonia, COVID-19-associated pneumonia was associated with higher NEFAs, lower apolipoprotein E and lower high-density lipoprotein cholesterol concentrations, independently of body mass index, sequential organ failure (SOFA) score, and C-reactive protein levels. NEFAs and PUFAs concentrations were negatively correlated with the number of ventilator-free days. Among hospitalized patients with severe pneumonia, COVID-19 is independently associated with higher NEFAs (mainly linoleic and arachidonic acids) and lower apolipoprotein E and HDL concentrations. These features might act as mediators in COVID-19 pathogenesis and emerge as new therapeutic targets. Further investigations are required to define the role of NEFAs in the pathogenesis and the dysregulated immune response associated with COVID-19.

Trial registration: NCT04435223.

## Introduction

Severe acute respiratory syndrome coronavirus 2 (SARS-CoV-2), which causes coronavirus disease 2019 (COVID-19), recently emerged as a major threat for human health. In its severe form, COVID-19 often leads to severe acute respiratory distress syndrome (ARDS). It also has specific features and outcomes that suggest a unique immunopathogenesis. A high proportion of obese patients has been reported among severe COVID-19 cases, and obesity has been associated with disease severity^1–3^. Obesity is characterized by metabolic disorders. We therefore assume that specific metabolic disorders rather than obesity per-se contribute to the severity of COVID-19^4^. In this context, lipid metabolism is of particular interest. Indeed, obesity is associated with impaired lipid metabolism (i.e. increased plasma triglyceride levels, decreased low density lipoprotein (LDL) and high density lipoprotein (HDL) concentrations^5^, and disturbed lipolysis and fatty acid metabolism^6^). Lipoproteins were previously shown to be involved in inflammation and innate immunity^7^. During SARS-CoV-2 infection, it was recently reported that lipid metabolism is dysregulated^8–11^, and lipid abnormalities were associated with poorer prognoses^12,13^. It has also been demonstrated that HDL-scavenger receptor B type 1 (SR-B1) facilitates SARS-Cov2 entry into ACE2-expressing cells^14^. Furthermore, a pocket for linoleic acid has been described on SARS-CoV-2 spike protein and linoleic acid appeared to stabilized SARS-CoV-2 spike protein in its closed form, resulting in reduced ACE2 interaction in vitro^15^. In addition, lipids are bioactive molecules that can act as inflammatory mediators^16^. Thus, it can be hypothesized that dysregulation of lipid metabolism influences virus pathogenicity and participate in the specific immune response. The current study aims to investigate the contribution of blood lipids and plasma lipoprotein abnormalities in COVID-19 pathogenesis by comparing lipoprotein metabolism between non-COVID-19 and COVID-19 patients with severe pneumonia.

## Results

### Patient characteristics

Sixty-one patients were enrolled (34 in the non-COVID-19 group and 27 in the COVID-19 group). Bacterial, viral, or mixed etiologies were proven in 9 (26%), 10 (29%) and 3 (9%) patients, respectively, from the non-COVID-19 group. Patient characteristics are shown in Table 1. Mean age was lower in the COVID-19 group than in the non-COVID-19 group (62 ± 11 vs. 69 ± 12; *p* = 0.03). Diabetes and dyslipidaemia were not significantly different between the two groups. Obesity was more frequent in the COVID-19 group (52%; n = 14) than in the non-COVID-19 group (35%; n = 12) but the difference did not reach statistical significance. At inclusion, global severity (assessed by SOFA score) and respiratory severity, (assessed by PaO_2_:FiO_2_ ratio) were not significantly different between the two groups (*p* = 0.43 for both). However, we observed a higher proportion of ARDS (93% vs 62%; *p* < 0.01) and a lower proportion of septic shock (0% vs 32%; *p* < 0.01) among COVID-19 patients. In the biological parameters, we observed lower plasma concentrations of C-reactive protein (CRP), lactate and glucose in COVID-19 patients.

**Table 1:** Patient characteristics according to COVID-19 status.

	non-COVID-19 N = 34	COVID-19 N = 27	*p*
**Demographic data**			
Male, n (%)	27 (79%)	17 (63%)	0.15
Age (year)	69 (± 12)	62 (± 11)	0.03
BMI (kg/m^2^)	29 (**± **7)	31 (**± **8)	0.40
**Medical history**			
Active smoking, n (%)	9 (26%)	2 (7%)	0.06
Chronic kidney insufficiency (moderate to severe), n (%)	2 (6%)	1 (4%)	0.70
Cerebrovascular disease, n (%)	5 (15%)	3 (11%)	0.68
Cardiovascular disease, n (%)	12 (35%)	5 (19%)	0.15
Pulmonary disease, n (%)	11 (32%)	5 (19%)	0.22
Diabetes, n (%)	10 (29%)	11 (41%)	0.36
Insulin-based therapy, n (%)	1 (3%)	2 (7%)	0.58
Metformin, n (%)	7 (21%)	10 (37%)	0.16
Dyslipidemia, n (%)	14 (41%)	12 (44%)	0.80
Statin therapy, n (%)	11 (32%)	8 (30%)	1
Hypertension, n (%)	18 (53%)	16 (59%)	0.62
**Initial severity**			
SOFA score	7.3 (± 3.7)	6.7 (± 2.0)	0.43
PSI score	121 (± 37)	94 (± 27)	< 0.01
Septic shock, n (%)	11 (32%)	0	< 0.01
Invasive mechanical ventilation, n (%)	23 (68%)	23 (85%)	0.11
ARDS at inclusion, n (%)	21 (62%)	25 (93%)	< 0.01
PaO_2_/FiO_2_ mmHg	126 (± 54)	136 (± 50)	0.43
**Biology**			
CRP (mg/l)	275 (± 149)	173 (± 63)	< 0.01
PCT (µg/l)	34 (± 63)	3 (± 7)	< 0.01
AST (UI/l)	89 (± 95)	86 (± 55)	0.90
ALT (UI/l)	49 (± 46)	70 (± 54)	0.11
Bilirubin (µmol/l)	15 (± 8)	12 (± 6)	0.13
Albumin (g/l)	23 (± 4)	23 (± 2)	0.11
NT-ProBNP (pg/ml)	5972 (± 7828)	2225 (± 229)	0.04
Serum creatinine (μmol/l)	137 (± 95)	90 (± 41)	0.01
Lactate (mmol/l)	2.7 (± 1.9)	1.7 (± 0.7)	< 0.01
**Glucose metabolism**			
Fructosamine (µmol/l)	493 (± 179)	516 (± 288)	0.73
Glycaemia (mmol/l)	16 (± 8)	12 (± 6)	0.02
Insulin (mU/l)	15 (± 25)	13 (± 12)	0.60
**Outcomes at 30 days**			
Thrombotic events, n (%)	2 (6%)	7 (26%)	0.03
Median days of mechanical ventilation	4 (0–15)	15 (7–22)	< 0.01
Median ICU length of stay (days)	12.5 (4–20)	20 (12–29)	0.03
Median hospital length of stay (days)	21 (13–30)	29 (20–30)	0.12
30-day mortality, n (%)	2 (6%)	1 (4%)	0.70

*BMI* body mass index, *SOFA* sequential organ failure assessment, *PSI* pneumonia severity index, *PaO*_*2*_ arterial oxygen partial pressure, *FiO*_*2*_ fraction of inspired oxygen, *RRT* renal replacement therapy, *CRP* C-reactive protein, *PCT* procalcitonin, *AST* aspartate transaminase, *ALT* alanine transaminase, *BNP* brain natriuretic peptide; ICU: intensive care unit.

Quantitative data are presented as mean ± SD or Median (IQR).

### Outcomes

COVID-19 patients had significantly higher duration of mechanical ventilation (15 [7–22] vs. 4 [0–15] days; *p* < 0.01). The 30-day mortality rate was 6% in the non-COVID-19 group and 4% in the COVID-19 group (*p* = 0.70) (Table 1).

### Plasma lipid profile is discriminating factor for non-COVID-19 vs COVID-19 patients

**Figure 1:**
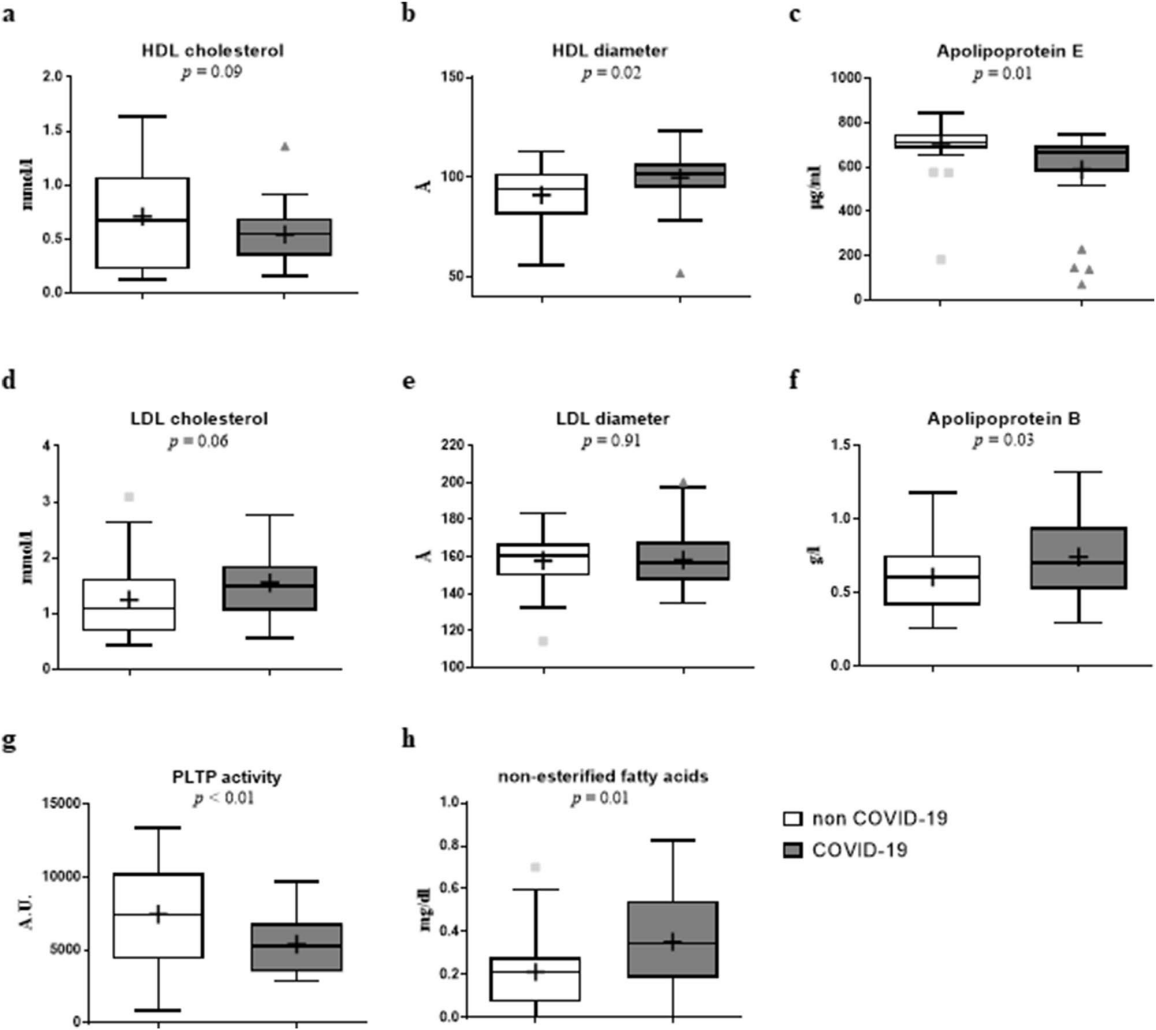
Boxplot representing selected lipid parameters depending on the COVID-19 status. Plasma concentration and lipoprotein diameter were measured in 34 non-COVID-19 and 27 COVID-19 patients with severe pneumonia. COVID-19 patients had significantly higher plasma concentrations of apolipoprotein B and NEFA and lower concentrations of Apolipoprotein E and phospholipid transfer protein. High density lipoprotein diameter was higher in COVID patients. HDL: High density lipoprotein; LDL: low density lipoprotein; PLTP: phospholipid transfer protein.

Plasma concentration of total cholesterol, phospholipids and triglycerides were not significantly different between non-COVID-19 and COVID-19 patients (Fig. 1, Table S1).

By contrast, COVID-19 patients showed higher HDL diameter (*p* = 0.02), lower ApoE concentrations (*p* = 0.01) and lower phospholipid transfer protein (PLTP) activity (*p* < 0.01) than non-COVID-19 patients, while the plasma levels of HDL cholesterol, apo A1 and cholesteryl ester transfer protein (CETP) activity were not significantly different (Fig. 1).

The study of LDL metabolism revealed higher concentrations of apoprotein B (ApoB) in COVID-19 patients (*p* = 0.03), while LDL cholesterol levels and particle size were not different in the two groups (Fig. 1).

Plasma triglyceride concentrations and VLDL size did not significantly differ between the two groups (*p* = 0.86 and *p* = 0.12), whereas the plasma concentration of non-esterified fatty acids (NEFAs) was significantly higher in COVID-19 patients (0.35 ± 0.22 vs. 0.21 ± 0.18 mg/dl; *p* = 0.01).

### Principal component analysis (PCA) identifies two patterns linking lipid metabolism and outcomes that enables distinguishing between non-COVID-19 and COVID-19 patients

**Figure 2:**
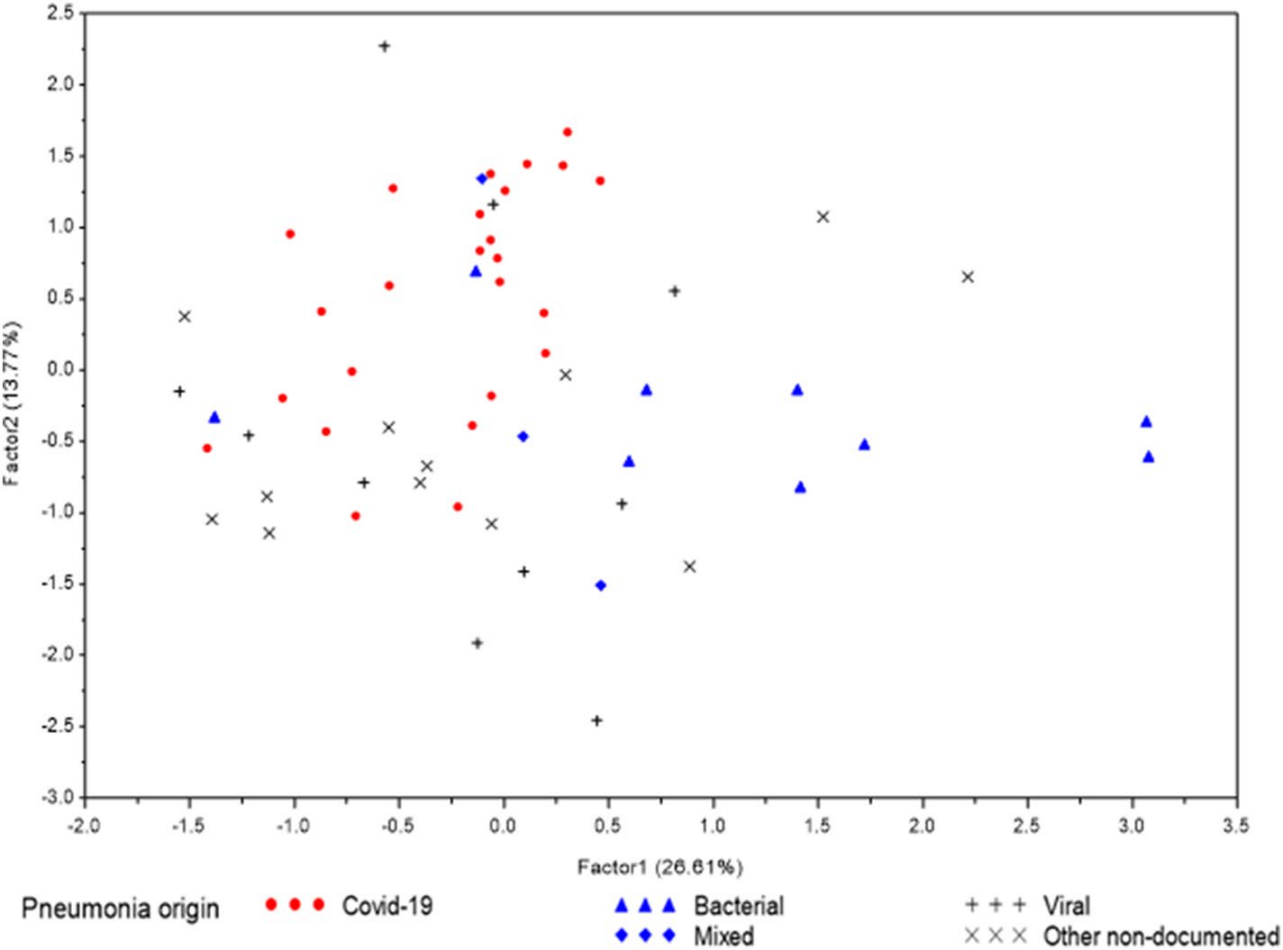
Factorial map for the primary component analysis. Patients are located according to their representation on each axis. Factor 1 appears to separate bacterial and viral pneumonia whereas factor 2 appears to separate COVID-19 and non-COVID pneumonia.

Using PCA, four factors were retained for interpretation. These factors preserve 58.4% of the total information contained in the 25 original correlated variables (Figure S1). The first component represents 26.6% of the data variability and was correlated to initial severity (SOFA score, pneumonia severity index (PSI)) and acute phase response (CRP). Levels of HDL cholesterol, Apolipoprotein A1 (ApoA1), HDL size, PLTP activity, LDL, ApoB, and LDL size were negatively correlated with this first component, while PLTP activity was positively correlated (Table 2). The second component represents 13.8% of the data variability and was correlated with outcomes (duration of mechanical ventilation, ICU length of stay). NEFAs concentrations were correlated with this second dimension, as were ApoA1 and HDL diameter (Table 2). The third component (10.1% of the data variability) represents the relationship between HDL, LDL and VLDL metabolism, but there was not a strong correlation with the outcome. The fourth component (7.8% of the data variability) is correlated with glucose metabolism and body mass index (BMI) (Table 2). The projection of patient data on the plan defined by factors 1 and 2 made it possible to discriminate COVID-19 from non-COVID-19 patients (Fig. 2). By plotting patients according to pneumonia etiology, we noticed that non-COVID-19 patients with pneumonia of proven bacterial origin were represented by high factor 1 values, while COVID-19 patients were represented by high factor 2 values and moderate factor 1 values (Fig. 2).

**Table 2:** Principal component analysis of clinical and lipid characteristics.

	Factor1	Factor2	Factor3	Factor4
HDL cholesterol	− 0.50	–	0.68	–
HDL diameter	− 0.67	0.32	–	–
Apolipoprotein A1	− 0.58	0.33	0.59	–
Apolipoprotein E	–	–	–	–
PLTP activity (A.U./min)	0.79	–	–	–
LDL cholesterol	− 0.69	–	–	0.34
LDL diameter	–	–	–	–
Apolipoprotein B	− 0.44	–	− 0.53	0.39
Total triglycerides	0.48	–	-0.58	–
VLDL diameter	− 0.50	–	0.48	–
Non-esterified fatty acid	–	0.33	–	–
Glycaemia	–	–	–	0.61
Insulin	–	–	–	0.77
BMI	–	–	–	0.64
PSI	0.52	–	0.46	–
SOFA	0.62	0.35	–	–
CRP	0.69	− 0.41	–	–
PCT	0.72	–	–	–
Creatininemia	0.66	–	0.31	–
NT-proBNP	0.69	–	0.38	–
Lactatemia	0.61	–	–	–
ICU length of stay	0.42	0.82	–	–
Duration of MV	0.42	0.85	–	–
Ventilator-free days	− 0.39	− 0.79	–	–
Hospital length of stay	0.46	0.67	–	–

Data are expressed as correlation coefficients. Values less than 0.3 are not edited.

*HDL* high density lipoprotein, *PLTP* phospholipid transfer protein, *LDL* low density lipoprotein, *VLDL* very low density lipoprotein, *BMI* body mass index, *PSI* pneumonia severity index, *SOFA* sequential organ failure assessment, *CRP* C reactive protein, *PCT* procalcitonin, *BNP* brain natriuretic peptide, *ICU* intensive care unit, *MV* mechanical ventilation.

### COVID-19 status is independently associated with NEFAs, HDL cholesterol and ApoE

Multiple linear regression showed that COVID-19 status was associated with high plasma NEFAs, low HDL cholesterol levels and low ApoE plasma concentrations, independently from body mass index (BMI), SOFA score and plasma CRP (Table 3). In COVID-19 patients, we estimated a proper excess of 0.12 ± 0.06 mg/dl for NEFAs (*p* = 0.04), and reductions of 0.36 ± 0.10 mmol/L in HDL cholesterol (*p* < 0.01) and 116.05 ± 45.49 ug/ml in Apolipoprotein E (ApoE) concentrations (p = 0.01). LDL cholesterol, HDL diameter and PLTP activity were independently associated with CRP concentration but not with COVID-19 status (Table 3).

**Table 3:** Factors associated with lipid parameters in 61 patients with severe pneumonia (multivariate linear regression).

Outcome variable effect	HDL cholesterol (mmol/l)			HDL diameter			Apo E (ug/ml)			LDL cholesterol (mmol/l)			PLTP activity (A.U)			NEFA (mg/dl)		
	Mean diff	± SE	*p*	Mean diff	± SE	*p*	Mean diff	± SE	*p*	Mean diff	± SE	*p*	Mean diff	± SE	*p*	Mean diff	± SE	*p*
COVID-19 (Yes vs No)	** − 0.36**	**0.10**	** < 0.01**	1.95	3.22	0.55	** − 116.05**	**45.49**	**0.01**	0.087	0.16	0.58	− 187.65	562.23	0.74	**0.12**	**0.056**	**0.04**
BMI (+ 1 kg/m^2^)	0.0068	0.0062	0.20	0.34	0.20	0.10	-2.92	2.86	0.31	0.0073	0.0096	0.45	− 18.51	34.66	0.59	− 0.0019	0.0035	0.58
SOFA (+ 1 point)	− 0.0054	0.016	0.74	** − 1.15**	**0.50**	**0.03**	− 4.21	6.96	0.55	** − 0.072**	**0.024**	** < 0.01**	144.85	87.22	0.10	0.017	0.0087	0.05
CRP (+ 10 mg/L)	** − 0.015**	**0.0041**	** < 0.01**	** − 0.50**	**0.113**	** < 0.01**	− 0.52	1.78	0.77	** − 0.018**	**0.006**	** < 0.01**	**167.88**	**224.11**	** < 0.001**	− 0.0038	0.002	0.09
R^2^	29%			42%			14%			34%			61%			20%		

*SE* standard error, *BMI* body mass index, *SOFA* sequential organ failure assessment, *CRP* C reactive protein, *NEFA* non-esterifierd fatty acid, *LDL* low density lipoprotein, *HDL* high density lipoprotein, *PLTP* phospholipid transfer protein, *ApoE* apolipoprotein E. Significant differences (*p* < 0.05) are written in bold

### Non-esterified fatty acid profile is characterized by high poly unsaturated fatty acid (PUFA) levels in COVID-19 patients

**Figure 3:**
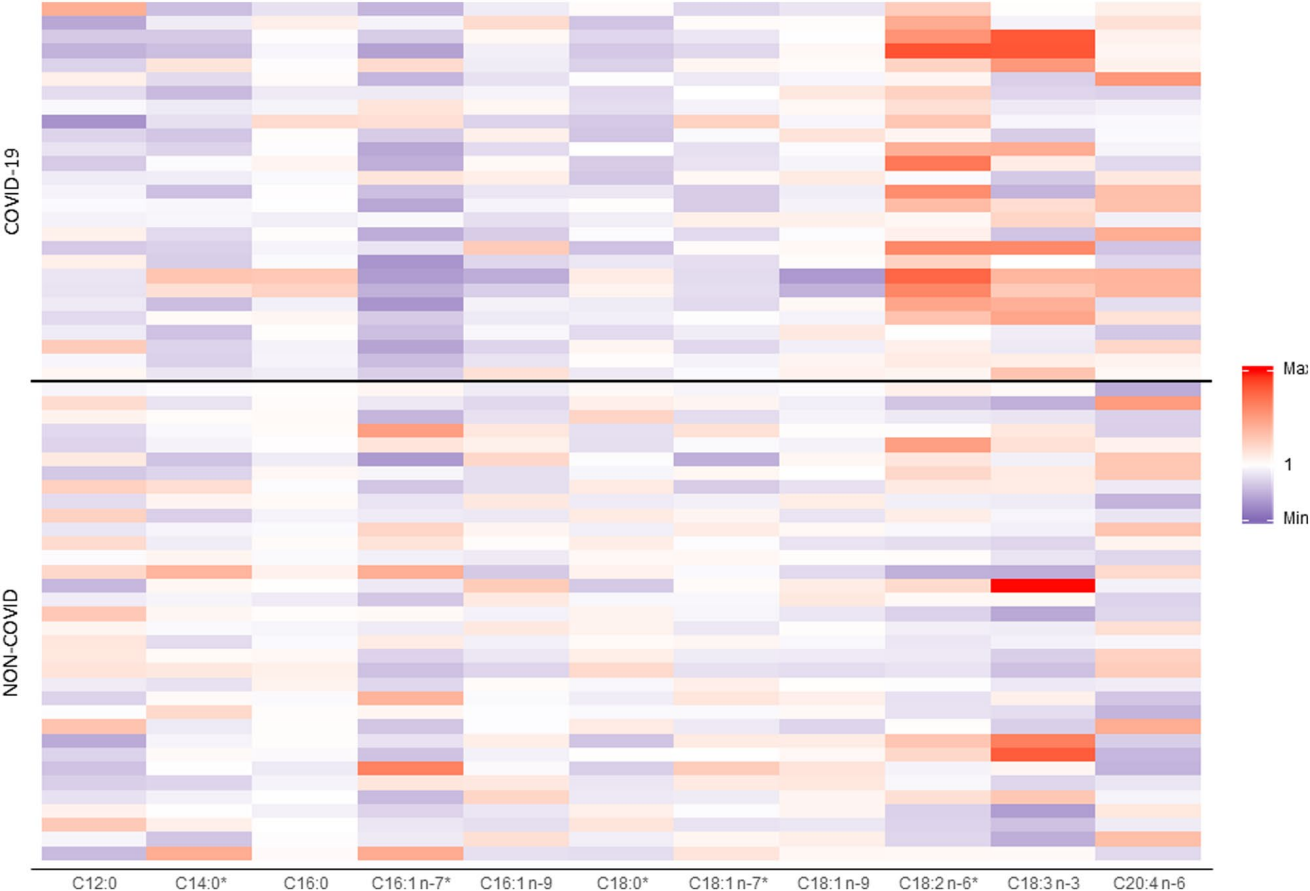
Heatmap of non-esterified fatty acid (NEFA) representation (as percentage normalized to the mean percentage in non-COVID-19 patients) according to COVID-19 status. Plasma concentration of NEFAs was measured in 34 non-COVID-19 and 27 COVID-19 patients with severe pneumonia. Rows represent patients. Colours represent the ratio between the percentage of NEFAs in the patient and the mean percentage of NEFAs in non-COVID-19 patients. In COVID-19 patients, linoleic acid appeared in red, reflecting a higher representation. *significant between-group differences (*p* < 0.05).

To gain further insights regarding the increase in plasma NEFAs, we performed a GC–MS profile of fatty acids (Fig. 3, Table S2). As expected, saturated and monounsaturated fatty acids (C16:0, C18:0 and C18:1) were the most represented fatty acids. However, the increase in NEFAs in COVID-19 patients was mainly driven by an increase in PUFA levels. Indeed among the lipid species with a plasma concentration higher than 10 nmol/mL, linoleic acid (C18:2 n-6) and arachidonic acid (C20:4 n-6) were significantly increased in COVID-19 patients (207 ± 109 vs. 113 ± 67 nmol/ml; *p* < 0.01 and 16 ± 6 vs 12 ± 5 nmol/ml *p* < 0.01, COVID-19 vs non-COVID-19 patients). Moreover, the relative proportion of linoleic acid among FAs was significantly higher in COVID-19 patients than in non-COVID-19 patients (12.8 ± 3.6 vs. 8.3 ± 2.3%; *p* < 0.01) (Fig. 2). However, sPLA2, Lp-PLA2, Leukotriene B4 and prostaglandin E concentrations did not differ significantly between the two groups (Table S1).

### Fatty acid metabolism cascade exploration

As phospholipase A2 (PLA_2_) mediates the release of fatty acids from membrane phospholipids, including linoleic and arachidonic acids, we measured both, secreted PLA_2_ (sPLA_2_) and lipoprotein-associated PLA_2_ (lp-PLA_2_) plasma concentrations. Plasma sPLA2 and lp-PLA_2_ concentrations were not significantly different between the two groups (respectively *p* = 0.93 and *p* = 0.25) (Table S1). In addition, we did not observe any correlation between plasma concentration of sPLA_2_ and lp-PLA_2_ and PUFAs (Table S3).

**Figure 4:**
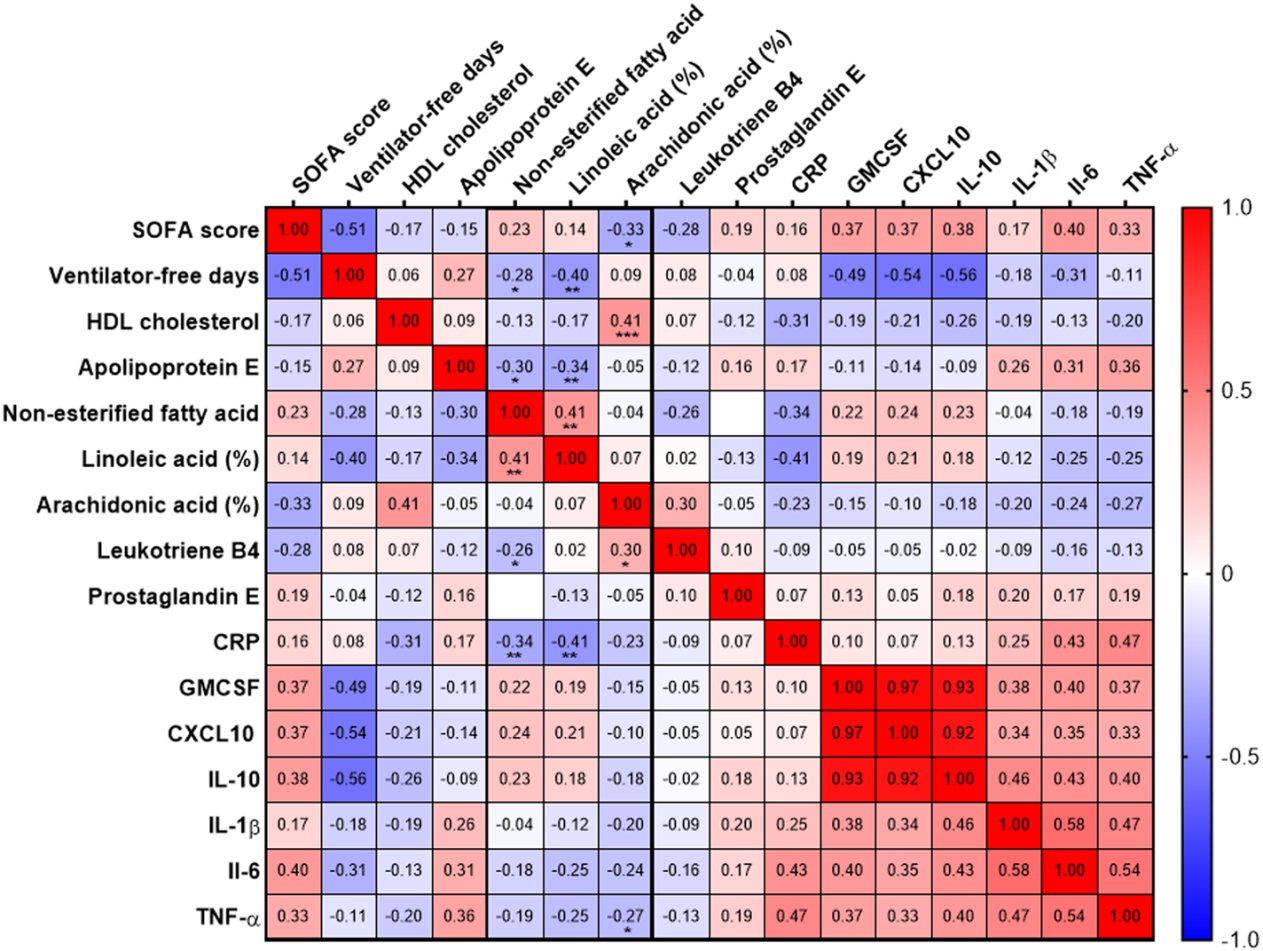
Correlations between NEFAs, PUFAs proportion, inflammation, and outcomes. Heatmap of the Spearman correlation (r) between lipids parameters, clinical severity and outcome, plasma cytokine concentrations. Spearman correlations**:** *p* < 0.05, *p < 0.01, **p < 0.001, ***between each variable. CRP: C-reactive protein; GMCSF: granulocyte–macrophage colony-stimulating factor; HDL: High density lipoprotein; IL: interleukin; SOFA: sequential organ failure assessment; TNF-α: tumor-necrosis factor alpha.

In addition, two final products of the arachidonic cascade were measured, namely leukotriene B4 and prostaglandin E2 (PGE2). We observed a correlation between arachidonic acid and leukotriene B4 concentrations (r = 0.302, *p* = 0.02) but not with PGE2 (r =  − 0.048, *p* = 0.9) (Fig. 4).

### Relationship between NEFAs, plasma cytokines and outcomes

Plasma NEFAs concentrations were marginally correlated with CXCL10, GMCSF, and IL-10 (respectively *p* = 0.064, *p* = 0.091, *p* = 0.075), but negatively correlated with CRP levels (r =  − 0.343, *p* = 0.007). In addition, linoleic and arachidonic acids proportions were marginally negatively correlated with IL-6 (*p* = 0.05 for both) and TNF-α concentrations (respectively *p* = 0.05 and *p* = 0.04) (Fig. 4).

Plasma NEFAs concentrations and linoleic acid proportion were inversely correlated with the number of ventilator-free days (respectively r =  − 0.279, *p* = 0.029 and r =  − 0.404, *p* = 0.001) (Fig. 4).

## Discussion

In patients with severe pneumonia, we report here an independent association between lipid metabolism disorders and COVID-19. Multivariate analysis showed that high NEFAs, low ApoE and low HDL cholesterol concentrations are significantly associated with COVID-19 status, independently of body mass index, baseline severity (SOFA score) and acute phase response (CRP level). The characterization of NEFAs highlighted an increase in polyunsaturated fatty acids (mainly linoleic and arachidonic acids). The concentrations of NEFAs, and particularly linoleic acids, were correlated with the duration of the mechanical ventilation.

COVID-19 pneumonia has unique features when compared with pneumonia from other origins, and it displays a specific inflammatory pathogenesis^17–19^. The beneficial effect of dexamethasone in the most severe forms of COVID-19 argues in favor of a dysregulated immune response that mediates lung injury and outcome^20^. We recently identified a unique cytokine response in COVID-19 patients with severe pneumonia with higher plasma GM-CSF and CXCL10 (a Th-1 chemokine) in COVID-19 patients, that were independently associated with the longer duration of mechanical ventilation^21,22^. Obesity has been described as a factor of poor prognosis in COVID-19 patients and associated with disease severity^1,2^. In various medical settings, obese patients display immune dysregulation (e.g. Th1 hyperpolarization) and metabolic disorders, and there is an obvious relationship between the two^23^. We assumed that metabolic disorders may contribute to the pathophysiology of COVID-19. In the present study, although obesity was highly frequent in COVID-19 patients (52% of patients), BMI, diabetes, dyslipidaemia and plasma fructosamine levels did not significantly differ between non-COVID-19 and COVID-19 patients. Therefore, obesity has a similar frequency in severe pneumonia regardless of the origin and was not likely to acts as a confounding factor between the 2 groups of patients.

Sepsis is associated with profound modifications in HDL (dramatic reduction in HDL-cholesterol, extensive remodelling of HDL particles) and LDL metabolism (lower LDL cholesterol concentrations)^24^. However, the mechanisms underlying this decrease are poorly described. PLTP activity is known to increase during systemic inflammation^25^. HDL and LDL were found to be protective in patients with sepsis^26^. The lower PLTP activity we observed in COVID-19 patients is consistent with the lower proportion of extra-pulmonary organ dysfunction (septic shock, lactatemia, RRT and creatininemia) and the lower acute phase response (CRP levels). With multivariate analyses, negative correlations between CRP and both HDL and LDL cholesterol levels were also consistent with these clinical settings. By contrast, low HDL and ApoE concentrations were independently associated with COVID-19 status. Interestingly, HDL particles are endowed with immune-modulatory properties partly through interaction with lipid rafts^27^ and HDL-scavenger receptor B type 1 (SR-B1) has been demonstrated to facilitate SARS-CoV-2 entry into ACE2-expressing cells^14^. Consequently, we assume that the more pronounced drop in HDL associated with COVID-19 origin could be the consequence of a higher consumption of HDL particles and/or the upregulation of SR-B1 expression. However, as in the ApoCOVID study, we did not find any association between HDL concentrations and outcomes^11^.

Then, we depict here higher plasma PUFAs concentrations (higher C18:2 n-6 and C20:4 n-6) and a higher proportion of PUFAs among NEFAs in COVID-19 patients as compared to non-COVID-19 patients. High plasma NEFAs concentrations (including PUFAs) were previously reported in COVID-19 patients as compared to healthy patients, independently of the acute phase response^28^. However, to our knowledge, there has not yet been a comparison between patients with severe pneumonia according to COVID-19 status. This absolute and relative increase in PUFAs supports a specific alteration of the fatty-acid metabolism of COVID-19 patients. PUFAs are precursors of eicosanoids and platelet activating factor. Such metabolite production could contribute to the dysregulated immune response and coagulopathy that characterize COVID-19. For example, MAPK activation has previously been suggested to activate cytosolic PLA_2_, which in turn increases thromboxane and platelet hyperactivity (31). These recent data support the hypothesis that PUFAs and the PLA_2_ pathway are potential key players in the pathogenesis of COVID-19. In our study, we did not find any correlation between the two plasmatic forms of PLA_2_ (namely s-PLA_2_ and lp-PLA_2_) and PUFAs, but we do not rule out an increase in cytosolic PLA_2_ expression and activity in COVID-19 patients, that we were unable to measure. However, we found a marginal positive correlation between NEFAs (and linoleic acid) and the three cytokines previously identified in higher concentrations and associated with poorer outcomes in COVID-19 patients^21^. That suggests the possible involvement of NEFAs in the specific immune response associated with COVID-19. Interestingly, SARS-CoV-2 comprises a high-affinity pocket that specifically accretes linoleic acids, modifying ACE_2_ interaction in-vitro^15^. The interaction between linoleic acids and SARS-CoV-2 represents an emerging appealing therapeutic target that requires further investigation. The correlations between NEFAs concentrations (and linoleic acids proportion) and the longer duration of mechanical ventilation we observed in COVID-19 strengthens these hypotheses.

To date, only corticosteroids have been proved to be effective in reducing mortality in severe COVID-19^20^, strengthening the host-immune response as one of the most promising therapeutic targets. Since corticosteroids have many side effects, targeted therapies likely to dampen the dysregulated immune response in COVID-19 are urgently needed. Our data highlight NEFAs as a potential driver of this dysregulated immune response that could drive poorer outcome. Restoring lipid homeostasis could represent a promising approach likely to dampen some features of the dysregulated immune response in COVID-19, and as a consequence outcomes. For this purpose, PLA-2 inhibitor is a potential candidate. In unselected patients with severe sepsis, PLA-2 inhibitors was shown to be ineffective in reducing mortality^29^. However, as for sepsis, personalized medicine with targeted immunomodulatory therapy, guided by immune-related biomarkers is mandatory to deal with the heterogeneity of the host-immune response^30^. Modulating lipid metabolism, using lipid-associated biomarkers (e.g. NEFAs concentrations) for decision making could represent a promising therapeutic strategy that deserve to be investigated in COVID-19.

There are some limitations in our work. Because this is an observational study, only association and not causality can be inferred. Statistical analysis may suffer from a lack of power since only 61 patients were included, but this was compensated, at least in part, by the strong observed differences and the use of several statistical methods including PCA and multivariate analysis. However, given this small sample size, our results need to be confirmed in larger cohorts. At this stage, the comparison of fatty acids metabolism and immune response between non-COVID-19 and COVID-19 patients with severe pneumonia is still scarce in the literature. We used CRP as a surrogate for acute phase response. However, CRP is also a known acute phase reactant^31^. Finally, we used plasma collection, and cytosolic PLA_2_ could not be measured to ascertain of the origin of plasma NEFAs.

In conclusion, COVID-19 was independently associated with high concentrations of polyunsaturated NEFAs and low concentrations of apolipoprotein E and HDL. These parameters may be key mediators of the COVID-19 pathogenesis and potential therapeutic targets. Further investigations are required to explore the role of cytosolic phospholipase A2 and PUFAs in the dysregulated immune response and the pathogenicity of COVID-19.

## Methods

### Design

This ancillary study of the ongoing prospective Lymphonie study (ClinicalTrials.gov NCT03505281) was registered in ClinicalTrials.gov (NCT04435223)^21^. The study fulfilled legal and ethics requirements: Approval was obtained from the CPP (Full name: *Comité de Protection des Personnes* SUD MEDITERRANEE V (reference 17.092); Registration number: 2017-A03404-49) for the original study and an amendment was obtained to include supplementary patients with severe COVID-19. All subjects (or their legal representatives) received written information and provided their consent to participate. The present report was drafted in line with the STROBE statement^32^.

### Patients

Patients were enrolled in this prospective monocentric cohort if they fulfilled the criteria for the Lymphonie study (previously presented on the original report^21^) : severe community-acquired pneumonia (CAP): (1) pneumonia (≥ 2 acute signs including cough, purulent sputum, dyspnea, chest pain, temperature < 35 °C or ≥ 38.5 °C, and novel radiological pulmonary infiltrate); (2) at least two criteria of poor prognosis according to the quick-SOFA score (systolic blood pressure ≤ 100 mm Hg, respiratory rate ≥ 22, Glasgow score < 15) and/or the need for mechanical ventilation (MV) and/or vasopressors; and (3) diagnosed within 48 h following admission. Non-inclusion criteria were: age < 18 years, pregnancy, person subject to a measure of legal protection, decision to limit care, known immune deficiency, chronic disorder known to cause deep lymphopenia (Cirrhosis, lympho or myeloproliferative syndrome, solid cancer or active systemic lupus), hospitalization for sepsis in the past 3 months. Non-COVID-19 CAP patients were included until February 20, 2020, one month before the COVID-19 pandemic started in Burgundy, France. COVID-19 patients were eligible if they tested positive for SARS-CoV-2 by reverse transcriptase-polymerase chain reaction on one respiratory sample. Patient with no available plasma sample were excluded from this analysis.

### Study procedures

Ethylenediaminetetraacetic acid blood was obtained after inclusion of the patient and within 48 h of hospital admission. Plasma was collected in the biological resource center (CRB Ferdinand Cabanne; NF S96-900 certification), centrifuged at 2000 ×g for 10 min at 4 °C and stored at  − 80 °C.

### Variables of interest, clinical outcomes, and data collection

Clinical and biological parameters, severity scores (Sequential Organ Failure Assessment (SOFA) (9) and the Pneumonia Severity Index (PSI) (11)) were calculated at the time of inclusion. Obesity was defined as body mass index (BMI) higher than 30. ARDS was defined according to the Berlin definition^33^. Septic shock was defined according to the third international consensus definitions for sepsis and septic shock^34^. Clinical outcomes were recorded up to 30 days after the hospital admission: 30-day mortality, number of hospital-, ICU-, and ventilator-free days. The ‘ventilator-free days’ outcome was defined as the number of days alive from day 1 of severe pneumonia to day 30 during which the patient was breathing without MV. Acute phase response is the systemic response to tissue damage^35^. We used CRP concentrations as a surrogate for APR because it is recognized as the prototype for acute phase reactant^31^.

### Biological judgment criteria

The primary judgment criterion was cholesterol concentration. Secondary judgment criteria were HDL metabolism parameters (HDL cholesterol, HDL size, ApoA1, ApoE, SAA), LDL metabolism parameter (LDL cholesterol, LDL size, ApoB), VLDL parameters (triglyceride concentration, VLDL size), NEFAs, lipid peroxydation and lipid transfer protein activity (PLTP and CETP activity).

### Measurement of lipid parameters

Total cholesterol concentration, total triglyceride concentration, high density lipoprotein cholesterol concentration (HDL-c) and low-density cholesterol concentration (LDL-c) were determined by colorimetric enzymatic methods. LDL-c and HDL-c were determined by direct methods. Apolipoprotein A1 (ApoA1) and B (ApoB) were measured by immunonephelemetry. All these parameters were determined on Dimension Vista analyzers using dedicated kits (SIEMENS).

Apolipoprotein E (ApoE) was measured by enzyme-linked immunosorbent assay (ELISA) (THERMO FISHER SCIENTIFIC INC., Massachusetts). Serum amyloid A was measured by immunoassays using the proQuantum method (THERMO FISHER SCIENTIFIC INC., Massachusetts). Lipid peroxidation was measured by fluorescence using the Tbars method (BIOASSAY SYSTEMS, Hayward, USA).

### Lipoprotein size determination

The different fractions of plasma were separated according to their density by ultracentrifugation (TLA100.2 rotor, BECKMAN, Palo Alto, CA). Densities were adjusted by adding appropriate volumes of potassium bromide solution. Three consecutive ultra-centrifugations were conducted at the appropriate densities in order to separate the plasma into 4 fractions: the triglyceride-rich fraction (*d* < 1.006), the LDL fraction (1.006 < *d* < 1.063), the HDL fraction (1.063 < *d* < 1.21) and the lipoprotein-free fraction (*d* > 1.21).

Triglycerides, cholesterol (free and total) and phospholipids in lipoproteins fractions isolated by ultracentrifugation were measured enzymatically by colorimetry with commercially available kits (DIASYS DIAGNOSTIC SYSTEMS GMBH, Holtzheim, Germany) according to manufacturer’s instructions. Lipoprotein size in isolated fractions was calculated based on the lipid composition as follows^36^: lipoprotein diameter (Å) = 20 * (radius of surface + radius of core); radius of surface being equal to 2.05 nm; radius of core being equal to 3 * [1.556*(moles%TG) + 1.068* (moles%CE)]/[0.685 (moles%PL) + 0.391 (mol%UC)].

### PLTP and CETP activity measurement

Phospholipid transfer protein (PLTP) activity was measured using a commercially available fluorescence activity assay (ROAR BIOMEDICAL INC., New York) according to the manufacturer’s instructions. Fluorescence measurements were performed over time on a Vicor^2^ multilabel counter (PERKIN ELMER). Phospholipid transfer activity was calculated from the slope of fluorescence increase between 1 and 30 min.

Cholesteryl ester transfer protein (CETP) activity was measured using a commercially available fluorescence activity assay (ROAR BIOMEDICAL INC., New York) according to the manufacturer’s instructions. Fluorescence measurements were performed over time on a Vicor^2^ multilabel counter. Cholesteryl ester transfer activity was calculated from the slope of fluorescence increase between 1 min and 2 h.

### Measurement of glucose metabolism parameters

Fructosamine and glucose concentrations were measured on total plasma by colorimetry using commercially available kits (CLINISCIENCES, Nanterre, France, for fructosamine and glucose). Insulin concentrations were measured by ELISA (MERCODIA AB, Sweden).

### Total NEFA concentrations

Total non-esterified fatty acids (NEFAs), fructosamine and glucose concentrations were measured on total plasma by colorimetry using commercially available kits (DIASYS DIAGNOSTIC SYSTEMS GMBH, HOLTZHEIM, Germany for NEFA).

### Characterization of NEFAs

The abundance of individual free fatty acids was determined by negative chemical ionization mode (NCI) GCMS in plasma. Deuterated fatty acids were from Cayman (BERTIN PHARMA, Montigny le Bretonneux, France). Chemicals of the highest grade available were purchased from SIGMA ALDRICH (Saint-Quentin Fallavier, France). LCMSMS quality grade solvents were purchased from FISCHER SCIENTIFIC (Illkirch, France). 25 µL plasma were spiked with 5 µL of free fatty acids internal mix containing 635 ng, 326 ng, 95 ng, of linoleic acid d4, arachidonic acid-d8, and DHA-d5, respectively. Samples were mixed with 1.2 ml of Dole’s reagent (Isopropanol/Hexane/Phosphoric acid 2 M 40/10/1 v/v/v). Free fatty acids were further extracted with 1 ml of Hexane and 1 ml of distilled water. Organic phase was collected and evaporated under vacuum. Fatty acids were analyzed as pentafluorobenzyl esters (PFB-FA esters) by NCI-GCMS as previously described (5). Calibration curves were obtained using linoleic acid (2–8 µg) arachidonic acid (1–4 µg) docosahexaenoic acid (0.4–16 µg) and docosapentaenoic acid (0.1–0.4 µg) extracted by the same method used for plasma. Linear regression was applied for calculations.

### Plasma PLA2, leukotriene B4 and prostaglandin E concentrations

Plasma sPLA2 and Lp-PLA2 concentrations were measured by ELISA (THERMOFISHER SCIENTIFIC, Massachusetts, United States). Plasma leukotriene B4 and prostaglandin E concentrations were measured by ELISA (CAYMAN CHEMICAL, MICHIGAN, United States).

### Plasma cytokines measurement

Plasma IL-1β, IL-6, IL-10, CXCL-10, GM-CSF and TNF-α, were quantified using a Luminex® Human Magnetic assay (R&D SYSTEMS, USA) to study the potential link between immune response and plasma lipids^21^.

### Statistical analyses

Collected data were described according to COVID-19 status. Continuous variables were expressed as means + /− standard deviations (SDs) or medians and interquartile ranges (IQRs) according to their distribution. Categorical variables were described as frequencies and percentages. Univariate analysis consists of comparisons between variables according to COVID-19 status, performed using the chi-square test (or Fisher’s exact test when appropriate) for percentages, the Student’s t-test for means and the Wilcoxon Mann–Whitney test for medians and IQRs. Lipoprotein metabolism parameters with *p* < 0.05 for Student’s t-tests were presented as boxplots to visualize potential associations between these parameters and COVID-19 status.

For multivariate analysis, principal component analysis (PCA) was used first to identify potentially significant patterns among the 25 variables (supplementary material): lipoprotein metabolism parameters (13 variables) and the following 12 clinical and biological parameters: BMI, PSI, SOFA, Lactate, ICU and hospital length of stay, ventilation duration and free days at day 30, CRP, PCT, creatinine and NT-ProBNP. PCA identifies factors, called principal components, that induce the most variation in the overall data. These factors are uncorrelated and can be expressed as a linear combination of the correlated original variables (OV). We can inverse these formulas to express each OV as a linear combination of factors. Coefficients defining these linear combinations are interpreted as correlation coefficients. A positive (or negative) coefficient means that the OV is positively (or negatively) associated with the factor. An absolute value approaching 1 indicates that the original variable has a strong influence on the value of the factor, while an absolute value below 0.3 is weak. Each factor describes a percent variation of the OVs. In order to determine the number of components to retain, we used the scree plot test: the number of factors to retain corresponds to the break point between factors with large eigenvalues and those with small eigenvalues on the scree plot^37^ and the clinical interpretability of these factors^38^. The labeling was primarily descriptive and based on our interpretation of the factors structure for the OVs strongly associated with the factor. In addition, OVs data can be projected and plotted on the plans defined by the retained factors, which reduces the dimensionality of a large amount of potentially interrelated OVs into a smaller set of factors. This makes it possible to observe variations and strong patterns among patients in a new, two-dimensional space without losing important information^39^.

Spearman’s rank correlations were computed between clinical characteristics and pertinent lipoprotein metabolism parameters associated with COVID-19 status in univariate analysis comparison tests. Multivariable linear regression was then performed between the selected lipoprotein metabolism parameters as independent variables and the COVID-19 status as a principal explicative covariate. To account for potential confounders, we constructed models adjusted for BMI, CRP and SOFA score. Absence of autocorrelation and heteroscedasticity were assessed using the DW statistic and the White test^40^, respectively. Model variability explicative power was quantified using the R^2^ coefficient. Association results were expressed as mean differences ± SE. A *p*-value below 0.05 was considered statistically significant. Analyses were performed using SAS software (version 9.4, SAS Institute, Inc., Cary, NC, USA).

### Ethics approval and consent to participate

This ancillary study of the ongoing prospective Lymphonie study (ClinicalTrials.gov NCT03505281) was registered in ClinicalTrials.gov (NCT04435223). The study fulfilled legal and ethics requirements: Approval was obtained from the CPP (*Comité de Protection des Personnes* SUD MEDITERRANEE V; 2017-A03404-49) for the original study and an amendment was obtained to include supplementary patients with severe COVID-19. All subjects (or their legal representatives) received written information and provided their informed consent to participate.

## Supplementary Information

Supplementary Information.

## Data Availability

All relevant data are within the paper. Raw data are available after notification and authorization of the competent authorities. In France, all computer data (including databases, particular patient data) are protected by the National Commission on Informatics and Liberty (CNIL), the national data protection authority for France. CNIL is an independent French administrative regulatory body whose mission is to ensure that data privacy law is applied to the collection, storage, and use of personal data. As the database of this study was authorized by the CNIL, we cannot make available data without prior agreement of the CNIL.

## References

[1] Lemyze M (2020). Implications of obesity for the management of severe coronavirus disease 2019 pneumonia. Crit. Care Med..

[2] Gao F (2020). obesity is a risk factor for greater COVID-19 severity. Diabetes Care.

[3] Simonnet A (2020). high prevalence of obesity in severe acute respiratory syndrome coronavirus-2 (SARS-CoV-2) requiring invasive mechanical ventilation. Obesity.

[4] Stefan N, Birkenfeld AL, Schulze MB, Ludwig DS (2020). Obesity and impaired metabolic health in patients with COVID-19. Nat. Rev. Endocrinol..

[5] Klop B, Elte JWF, Cabezas MC (2013). Dyslipidemia in obesity: mechanisms and potential targets. Nutrients.

[6] Blaak EE (2003). Fatty acid metabolism in obesity and type 2 diabetes mellitus. Proc. Nutr. Soc..

[7] Navab M, Anantharamaiah GM, Fogelman AM (2005). The role of high-density lipoprotein in inflammation. Trends Cardiovasc. Med..

[8] Shen B (2020). Proteomic and metabolomic characterization of COVID-19 patient sera. Cell.

[9] Hu X, Chen D, Wu L, He G, Ye W (2020). Low serum cholesterol level among patients with COVID-19 infection in Wenzhou China. SSRN Electron. J..

[10] Wei X (2020). Hypolipidemia is associated with the severity of COVID-19. J. Clin. Lipidol..

[11] Tanaka S (2020). Lipoprotein concentrations over time in the intensive care unit COVID-19 patients: results from the ApoCOVID study. PLoS ONE.

[12] Fan J (2020). Low-density lipoprotein is a potential predictor of poor prognosis in patients with coronavirus disease 2019. Metabol. Clin. Exp..

[13] Wang, G. *et al.* Low high-density lipoprotein level is correlated with the severity of COVID-19 patients. 10.21203/rs.3.rs-34659/v1 10.1186/s12944-020-01382-9 PMC7475024 32892746

[14] Wei C (2020). HDL-scavenger receptor B type 1 facilitates SARS-CoV-2 entry. Nat. Metab..

[15] Toelzer C (2020). Free fatty acid binding pocket in the locked structure of SARS-CoV-2 spike protein. Science.

[16] Tam VC (2013). Lipidomic profiling of bioactive lipids by mass spectrometry during microbial infections. Semin. Immunol..

[17] Helms J (2020). High risk of thrombosis in patients with severe SARS-CoV-2 infection: a multicenter prospective cohort study. Intensive Care Med..

[18] Marini JJ, Gattinoni L (2020). Management of COVID-19 respiratory distress. JAMA J. Am. Med. Assoc..

[19] Luciani M (2020). Coinfection of tuberculosis pneumonia and COVID-19 in a patient vaccinated with Bacille Calmette-Guérin (BCG): case report. SN Compr. Clin. Med..

[20] Recovery Collaborative Group (2020). Dexamethasone in hospitalized patients with Covid-19: preliminary report. N. Engl. J. Med..

[21] Blot M (2020). The dysregulated innate immune response in severe COVID-19 pneumonia that could drive poorer outcome. J. Transl. Med..

[22] Blot M (2020). CXCL10 could drive longer duration of mechanical ventilation during COVID-19 ARDS. Crit. Care.

[23] Viardot A (2012). Obesity is associated with activated and insulin resistant immune cells. Diabetes. Metab. Res. Rev..

[24] van Leeuwen HJ (2003). Lipoprotein metabolism in patients with severe sepsis. Crit. Care Med..

[25] Barlage S (2001). ApoE-containing high density lipoproteins and phospholipid transfer protein activity increase in patients with a systemic inflammatory response. J. Lipid Res..

[26] Murch O, Collin M, Hinds CJ, Thiemermann C (2007). Lipoproteins in inflammation and sepsis I. Basic science. Intensive Care Med..

[27] Norata GD, Pirillo A, Ammirati E, Catapano AL (2012). Emerging role of high density lipoproteins as a player in the immune system. Atherosclerosis.

[28] Thomas, T. *et al.* COVID-19 infection alters kynurenine and fatty acid metabolism, correlating with IL-6 levels and renal status. *JCI Insight* **5**, (2020). 10.1172/jci.insight.140327 PMC7453907 32559180

[29] Zeiher BG (2005). LY315920NA/S-5920, a selective inhibitor of group IIA secretory phospholipase A2, fails to improve clinical outcome for patients with severe sepsis*. Crit. Care Med..

[30] van der Poll T, van de Veerdonk FL, Scicluna BP, Netea MG (2017). The immunopathology of sepsis and potential therapeutic targets. Nat. Rev. Immunol..

[31] Gewurz H, Mold C, Siegel J, Fiedel B (1982). C-reactive protein and the acute phase response. Adv. Intern. Med..

[32] von Elm E (2007). The Strengthening the reporting of observational studies in epidemiology (STROBE) statement: guidelines for reporting observational studies. PLoS Med..

[33] Ranieri VM (2012). Acute respiratory distress syndrome: The Berlin definition. JAMA - J. Am. Med. Assoc..

[34] Singer M (2016). The third international consensus definitions for sepsis and septic shock (Sepsis-3). JAMA.

[35] Baumann H, Gauldie J (1994). The acute phase response. Immunol. Today.

[36] Kuksis A, Breckenridge WC, Myher JJ, Kakis G (1978). Replacement of endogenous phospholipids in rat plasma lipoproteins during intravenous infusion of an artificial lipid emulsion. Can. J. Biochem..

[37] Cattell RB (1966). The scree test for the number of factors. Multivariate Behav. Res..

[38] Cattell RB, Vogelmann S (1977). A comprehensive trial of the scree and kg criteria for determining the number of factors. Multivariate Behav. Res..

[39] Jolliffe, I. T. Principal Components in Regression Analysis. in 129–155 (Springer, New York, NY, 1986). 10.1007/978-1-4757-1904-8_8.

[40] White H (1980). A heteroskedasticity-consistent covariance matrix estimator and a direct test for heteroskedasticity. Econometrica.

