# Supplementary material for: High plasma concentration of non-esterified polyunsaturated fatty acids is a specific feature of severe COVID-19 pneumonia
Source: Sci Rep. 2021 May 24;11:10824. doi: 10.1038/s41598-021-90362-9 (PMC8144366; doi:10.1038/s41598-021-90362-9)
Supplement: Supplementary file 1 — Supplementary Information. [file 41598_2021_90362_MOESM1_ESM.docx]

**High plasma concentration of non-esterified polyunsaturated fatty acids is a specific feature of severe COVID-19 pneumonia**

Maxime Nguyen^1-2^, MD, Abderrahmane Bourredjem^3^, MSc, Lionel Piroth^3-4^, MD,PhD, Bélaïd Bouhemad^1-2^, MD, PhD, Antoine Jalil^2^, MSc, Gaetan Pallot^2^, MSc, Naig Le Guern^2^, Charles Thomas^2^, PhD, Thomas Pilot^2^, MSc, Victoria Bergas^6^, Hélène Choubley^6^, Jean-Pierre Quenot^2,3,5^, MD, PhD, Pierre-Emmanuel Charles^2-5^, MD, PhD, Laurent Lagrost^2^, PhD, Valerie Deckert^2^, PhD, Jean-Paul Pais de Barros^2-6^, PhD, Pierre-Grégoire Guinot^1-2^, MD, PhD, David Masson^2-7^,Pharm.D., PhD, Christine Binquet^3^, PhD, Thomas Gautier^2^, PhD, Mathieu Blot^2-4^, MD, PhD,

for the LYMPHONIE study group

-

**Supplementary data**

**Table S1.** Lipid metabolism according to COVID-19 status

|  | **non-COVID-19** | **COVID-19** | ***p-value*** |
| --- | --- | --- | --- |
|  | **N= 34** | **N=27** |  |
| Total cholesterol (mmol/l), mean ±SD | 2.83 ±1.02 | 2.86 ±0.75 | 0.89 |
| Total phospholipid (mg/dl), mean ±SD | 1.57 ±0.39 | 1.56 ±0.32 | 0.94 |
| Total triglycerides (mmol/l), mean ±SD | 2.06 ±1.66 | 2.11 ±0.55 | 0.86 |
| **HDL parameters** |  |  |  |
| HDL cholesterol (mmol/l), mean ±SD | 0.71 ±0.49 | 0.54 ±0.27 | 0.09 |
| HDL diameter (Å), mean ±SD | 91 ±14 | 100 ±14 | **0.02** |
| Apolipoprotein A1 (g/l), mean ±SD | 0.83 ±0.37 | 0.77 ±0.21 | 0.37 |
| Serum amyloid protein (pg/ml), mean ±SD | 87 ±126 | 45 ±44 | 0.08 |
| Apolipoprotein E (µg/ml), mean ±SD | 700 ±108 | 589 ±200 | **0.01** |
| **Lipid transfer protein activities** |  |  |  |
| PLTP activity (A.U./min), mean ±SD | 7437 ±3484 | 5379 ±5242 | **< 0.01** |
| CETP activity (A.U./min), mean ±SD | 12 ±9 | 8 ±8 | 0.12 |
| **Apo-B containing lipoproteins** |  |  |  |
| LDL cholesterol (mmol/l), mean ±SD | 1.25 ±0.70 | 1.55 ±0.55 | 0.06 |
| LDL diameter (Å), mean ±SD | 158 ±14 | 158 ±15 | 0.91 |
| VLDL diameter (Å), mean ±SD | 329 ±111 | 296 ±42 | 0.12 |
| Apolipoprotein B (g/l), mean ±SD | 0.61 ±0.23 | 0.75 ±0.25 | **0.03** |
| **Fatty acid metabolism** |  |  |  |
| Non-esterified fatty acid (mg/dl), mean ±SD | 0.21 ±0.18 | 0.35 ±0.22 | **0.01** |
| sPLA2 (pg/ml), mean ±SD | 44905 ±23648 | 44375 ±24932 | 0.93 |
| Lp-PLA2 (ng/ml), mean ±SD | 47.6 ±146 | 17.1 ±21.3 | 0.25 |
| Leukotriene B4 (pg/ml), mean ±SD | 228 ±259 | 252 ±214 | 0.69 |
| Prostaglandin E (pg/ml), mean ±SD | 136 ±73.3 | 132 ±82.8 | 0.83 |
| Lipid peroxidation (detected by Tbars), n (%) | 8 (24%) | 6(22%) | 0.90 |

Footnotes: HDL: high density lipoprotein, Apo-B: Apolipoprotein B, LDL: low density lipoprotein, VLDL: very low-density lipoprotein, PLTP: phospholipid transfer protein, CETP: cholesterol ester transfer protein

**Table S2.** Non-esterified fatty acids according to COVID-19 status.

| **NEFAs (nmol/mL)** |  | **non-COVID-19**  **N=34** | **COVID-19**  **N=27** | ***p-value*** |
| --- | --- | --- | --- | --- |
| C12:0 |  | 148 ±15.9 | 161 ±36.3 | 0.10 |
| C14:0 |  | 19.0 ±9.68 | 18.7 ±8.48 | 0.89 |
| C16:0 |  | 340 ±128 | 432 ±222 | 0.06 |
| C16:1 n-9 |  | 4.00 ±2.03 | 4.46 ±2.16 | 0.40 |
| C16:1 n-7 |  | 44.6 ±29.8 | 38.6 ±32.6 | 0.46 |
| C18:0 |  | 120 ±24.2 | 125 ±31.0 | 0.50 |
| C18:1 n-9 |  | 434 ±210 | 514 ±231 | 0.17 |
| C18:1 n-7 |  | 36.6 ±18.6 | 40.5 ±25.5 | 0.51 |
| C18:2 n-6 |  | 113 ±66.9 | 207 ±109 | **<0.01** |
| C18:3 n-6 |  | 0.64 ±0.44 | 0.82 ±0.77 | 0.28 |
| C18:3 n-3 |  | 7.84 ±7.32 | 11.0 ±7.01 | 0.09 |
| C20:0 |  | 1.27 ±0.24 | 1.20 ±0.44 | 0.46 |
| C20:1 n-9 |  | 3.53 ±1.62 | 3.60 ±1.68 | 0.87 |
| C20:1 n-7 |  | 0.31 ±0.14 | 0.25 ±0.17 | 0.17 |
| C20:2 n-6 |  | 1.34 ±0.55 | 1.63 ±0.80 | 0.12 |
| C20:3 n-6 |  | 1.83 ±0.92 | 2.09 ±1.27 | 0.38 |
| C20:3 n-9 |  | 0.49 ±0.29 | 0.51 ±0.73 | 0.90 |
| C20:4 n-6 |  | 11.5 ±4.53 | 16.3 ±6.28 | **<0.01** |
| C20:5 n-3 |  | 1.37 ±0.69 | 1.37 ±0.79 | 0.99 |
| C22:0 |  | 0.15 ±0.05 | 0.15 ±0.08 | 0.88 |
| C22:1 n-9 |  | 0.41 ±0.20 | 0.36 ±0.18 | 0.29 |
| C22:1 n-7 |  | 0.02 ±0.01 | 0.02 ±0.01 | 0.46 |
| C22:4 n-6 |  | 1.29 ±0.66 | 1.50 ±0.89 | 0.32 |
| C22:6 n-3 |  | 4.68 ±2.37 | 6.04 ±2.37 | **0.03** |

Footnotes: Mean ±SD are represented for each variable.

NEFAs: non esterified fatty acids.

**Table S3.** Correlation between PLA2 and fatty acids

|  | **NEFAs** | **C18:2 n-6** | **C20:4 n-6** | **PGE2** | **LB4** |
| --- | --- | --- | --- | --- | --- |
| sPLA_2_ | -0.06 | 0.05 | 0.10 | -0.12 | -0.08 |
| lp-PLA_2_ | 0.00 | -0.13 | -0.07 | 0.01 | -0.09 |

Footnotes : NEFAs : non-esterified fatty acids; PGE2: Prostaglandin E2; LB4: Leukotriene B4; sPLA_2_: secreted phospholipase A_2_; lp-PLA_2_: lipoprotein associated phospholipase A_2_.

Results are presented as spearman correlation coefficients, p > 0.05 for all values.

**Figure S1.** Principal component analysis scree plot (retained dimension = 4)
